# The STRIDE (Strategies to Increase confidence, InDependence and Energy) study: cognitive behavioural therapy-based intervention to reduce fear of falling in older fallers living in the community - study protocol for a randomised controlled trial

**DOI:** 10.1186/1745-6215-15-210

**Published:** 2014-06-06

**Authors:** Steve W Parry, Vincent Deary, Tracy Finch, Claire Bamford, Neil Sabin, Peter McMeekin, John O’Brien, Alma Caldwell, Nick Steen, Susan L Whitney, Claire Macdonald, Elaine McColl

**Affiliations:** 1 Institute for Ageing and Health, Newcastle University, c/o Falls and Syncope Service, Royal Victoria Infirmary, Queen Victoria Road, Newcastle upon Tyne NE1 4LP, UK; 2 Department of Psychology, Northumbria University, Northumberland Road, Newcastle upon Tyne NE1 8ST, UK; 3 Institute of Health and Society, Newcastle University, Baddiley Clark Building, Richardson Road, Newcastle upon Tyne NE2 4AX, UK; 4 Newcastle University, c/o STRIDE Office, 3-4 Claremont Road, Newcastle upon Tyne NE2 4AE, UK; 5 Department of Psychiatry, University of Cambridge School of Clinical Medicine, Bradbury Centre, Level E4 Cambridge Biomedical Campus, Cambridge CB2 0SP, UK; 6 Age UK North Shields, 13 Saville Street West, North Shields NE29 6QP, UK; 7 University of Pittsburgh, 6035 Forbes Tower, Pittsburgh, PA 15260, USA

**Keywords:** Fear of falling, Falls, Elders, Community, Randomised controlled trial, Cognitive behavioural therapy, Complex intervention, Normalization process theory

## Abstract

**Background:**

Around 30% to 62% of older individuals fall each year, with adverse consequences of falls being by no means limited to physical injury and escalating levels of dependence. Many older individuals suffer from a variety of adverse psychosocial difficulties related to falling including fear, anxiety, loss of confidence and subsequent increasing activity avoidance, social isolation and frailty. Such ‘fear of falling’ is common and disabling, but definitive studies examining the effective management of the syndrome are lacking. Cognitive behavioural therapy has been trialed with some success in a group setting, but there is no adequately powered randomised controlled study of an individually based cognitive behavioural therapy intervention, and none using non-mental health professionals to deliver the intervention.

**Methods/Design:**

We are conducting a two-phase study examining the role of individual cognitive behavioural therapy delivered by healthcare assistants in improving fear of falling in older adults. In Phase I, the intervention was developed and taught to healthcare assistants, while Phase II is the pragmatic randomised controlled study examining the efficacy of the intervention in improving fear of falling in community-dwelling elders attending falls services. A qualitative process evaluation study informed by Normalization Process Theory is being conducted throughout to examine the potential promoters and inhibitors of introducing such an intervention into routine clinical practice, while a health economic sub-study running alongside the trial is examining the costs and benefits of such an approach to the wider health economy.

**Trial registration:**

Current Controlled Trials ISRCTN78396615

## Background

Falls are common, frequently devastating events for older people, with between 30% and 62% of older individuals falling per year [1,2]. Falls are responsible for considerable morbidity and mortality, with around 10% of falls resulting in fractures [1]. The cost of falls to the UK economy is estimated at £981 million [3], with more recent data showing that 0.07% to 0.20% of the gross domestic product and 0.85% to 1.5% of total healthcare expenditure in western economies was accounted for by falls and their consequences [4]. Adverse consequences of falls are by no means limited to physical injury and escalating levels of dependence. Many older individuals, both fallers and non-fallers, experience a variety of adverse psychosocial difficulties related to falling [5-15] including fear, anxiety, loss of confidence, and impaired self-efficacy (the self-perception of ability to perform within a particular domain of activities) [9,12] resulting in activity avoidance, social isolation and increasing frailty [5-15]. The umbrella term for these problems is ‘fear of falling’, a common and disabling problem in older individuals, found in between 3% and 85% of community-dwelling elders who fall, and up to 50% of those who have never fallen [7-9,15].

The optimal management strategy for fear of falling and its adverse physical and psychosocial sequelae is poorly understood. Much previous research has focused on physical treatments including home- and community-based exercise interventions, tai chi and multifactorial interventions aimed at reducing fall rates, with fear of falling reported as a secondary outcome in the majority of these studies [7]. A recent systematic review found 12 high-quality randomised controlled trials (RCTs) reporting effects on fear of falling in such studies, but only one primarily aimed at reducing fear of falling [15]. The interventions were conducted across a variety of settings, but home-based exercise, community tai chi and home-based multifactorial interventions all improved fear of falling [7], though a recent geriatric outpatient-based multifactorial intervention study found no such benefit [16].

While such physical interventions may be of benefit in selected populations, the profile of the disorder and its psychosocial complications suggest that well-designed psychological interventions may help ameliorate fear of falling more definitively. Several studies have examined an explicitly cognitive behavioural therapeutic (CBT) approach in fear of falling in community-dwelling elders, or used CBT techniques as part of a wider intervention strategy. Tennstedt *et al*.’s Matter of Balance study assessed the ability of an eight-session, 4-week group CBT with exercise instruction to improve fear of falling and related activity restriction [17]. Significant differences between intervention and control groups were seen in fear of falling as measured by the Falls Efficacy Scale (FES) [12] and activity during follow-up. The magnitude of improvement in FES scores attenuated over time, prompting the authors to suggest a booster session should be used in future studies and in clinical practice [17]. Clemson *et al.* similarly used what they described as a ‘small-group learning environment’ (though in practice, some of the methods used included CBTs) of 12 individuals per group for 2 hours per session over 7 weeks to improve self-efficacy and reduce falls [18]. The intervention incorporated a variety of learning strategies to facilitate behaviour change, including education regarding exercises to improve falls risks, medication and home environmental review and medication management [18]. There was a 31% reduction in falls (relative risk 0.69, 95% CI 0.50 to 0.96, *P* = 0.025) in the intervention group, though interestingly there was no corresponding change in FES scores [18]. More recently, Zijlstra and colleagues conducted a RCT of a multicomponent cognitive behavioural group intervention in older community-dwelling elders [19]. Five hundred and forty participants were drawn from a random sample of 7,431 individuals who reported (through self-report questionnaires) ‘at least some fear of falling’, though the precise method of assessment was not specified. Following randomisation, the intervention group underwent a structured 2-hour group CBT intervention based on the investigators’ previous work once weekly for 8 weeks, with booster sessions 6 months following the last session. A primary outcome was not specified, and while a power calculation was supplied based on a ‘difference of 2.5 points’, the authors fail to specify on which scale. The beginning of the trial predates widespread use of the FES-International version (FES-I) [20] (later advocated by the same group as the most appropriate measure for such studies [7]), instead using a single item question on fear of falling as well as an unspecified scale, likely to be the original FES from the description and reference supplied [19]. Other outcomes included perceived control over falling and daily activity as well as falls. There were no measures of physical function despite the prior evidence base suggesting improvement in fear of falling with the exercise-related measures as described above. All outcomes showed significant differences between control and intervention groups at 2 and 8 months follow-up, with between-group differences persisting at 14 months in fear of falling and perceived control over falling but not in the other outcome measures. There was a 30% attrition rate in the intervention group and 19.6% attrition rate in the control group [19]. The study intervention was carefully developed and grounded in CBT, but interpretation and application is hampered considerably by the lack of clarity on sample size calculation and outcome measures and the absence of generic quality-of-life measures and measures of physical functioning. Importantly, there is also no health economic analysis [19] to guide commissioners and providers of healthcare, crucial in this context because of the size of the clinical problem.

Fear of falling is thus a common, disabling and debilitating condition in older adults but the current understanding of its management is limited. There is a small evidence base to support the use of some physical therapies to improve the syndrome, and promising early data from a few studies supporting the use of psychological therapies, in particular CBT. The cognitive behavioural model [21] of a problem situation being maintained by an interaction between physiological, behavioural, cognitive and affective responses is paradigmatic for fear of falling, and offers the hope of a viable therapeutic option. Previous studies are hampered by the factors already described, while the issue of the economic viability of such a treatment has yet to be explored.

There is a need for many more trained cognitive behavioural therapists than are currently available; the development of a cognitive therapeutic package for the management of fear of falling that can be delivered routinely by non-specialist staff such as healthcare assistants (HCA) is vital if this common and debilitating condition is to be tackled effectively. CBT can be delivered by suitably trained non-psychotherapist staff [22,23], but to the best of our knowledge, this approach has not been attempted in a RCT in this context previously. In addition, only group interventions have been studied so far, with therapy delivered on a one-to-one basis yet to be tested in a fear of falling cognitive behavioural intervention study.

Understanding the dynamics of developing, delivering and trialling a novel intervention as a process is useful because it will contribute to understanding the professional and organisational factors that promote or inhibit adherence to treatment protocols and intervention delivery; and how practical and methodological problems are defined, understood and resolved by the project team in the course of the study. The need for understanding the dynamics of complex interventions [24], and undertaking process evaluation is now well understood [25]. Such work is important to underpin the transportability, workability, and integration of interventions into routine clinical practice. In the case of this trial, our aim is to collect longitudinal ethnographic data that will help us to understand the social processes and relationships that lead the intervention and trial to take a particular shape and direction. In earlier studies of trials and other interventions, May and Finch developed a robust explanatory model of normalization processes [26] that defines psychological and sociological mechanisms of behaviour and action that have been empirically demonstrated to be important in the implementation of complex interventions, and that have been revealed by evaluation in randomised controlled clinical trials. This approach is vital for the understanding and more widespread adoption of such an intervention.

In summary, we aim to develop a cognitive behaviour-based intervention to be delivered by HCAs on a one-to-one basis to community-dwelling older individuals attending falls services with an excessive or undue fear of falling and then to conduct a randomised controlled study of this intervention plus usual multidisciplinary care versus usual multidisciplinary care alone. Our study, incorporating a qualitative process evaluation and economic evaluation alongside a patient RCT, will answer definitively the question of whether an enhanced intervention to reduce the fear of falling for community-living older people would be effective and cost effective in reducing anxiety through its rigorous design and carefully chosen primary and secondary outcome measures, while assessing the cost and outcomes of such an intervention.

## Methods

**Figure 1:**
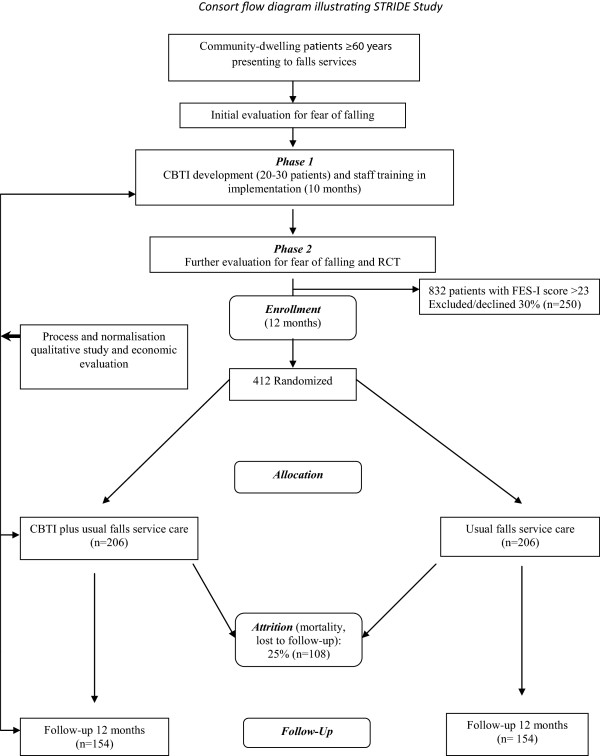
Consort flow diagram illustrating the STRIDE study.

The study is divided into two distinct phases, the first developing the novel cognitive behavioural therapy-based intervention (CBTI) and the second the RCT assessing the effectiveness of the intervention in reducing fear of falling. Qualitative and health economic studies straddle the two phases (Figure 1).

### Phase 1

Pilot study developing a cognitive behavioural model tailored to older patients [27] with fear of falling, based on the Medical Research Council (MRC) model of complex intervention development [28] and incorporating elements of previously successful CBT-based interventions [16-19]. Patients with significant fear of falling attending our community falls service were included in this phase of the study, and HCAs trained to deliver the intervention.

### Phase 2

Parallel-group patient RCT of the novel CBTI plus usual multidisciplinary care versus usual multidisciplinary care alone in patients with significant fear of falling attending multidisciplinary falls services. Patients are randomised in a 1:1 ratio, using a web-based system to ensure concealment of allocation, to intervention and control groups. Randomisation is stratified by gender (to avoid any potential sex differences in the response of older individuals to CBT-type interventions) and by whether the patient has been referred for strength and balance training. Study centre (random effect) and pain score (fixed effect) are also entered in to the randomisation system as stratification variables. Fear of falling is well documented in fallers from 40 years to extreme old age [6-10]; accordingly, we do not feel that stratification by age will add to the assessment of the intervention’s effectiveness; in addition, we can find no compelling evidence that older individuals respond to CBT differently. Our study is an individually randomised trial, delivered by three therapists (HCAs), and as such groups could theoretically be open to contamination (that is patients allocated to the control condition receiving CBTI). We believe that this risk is minimal, particularly at clinician level as the HCAs delivering the CBTI during Phase 2 will only be seeing the intervention group of patients and will not be part of the teams delivering usual care.

### Across project duration

A qualitative process evaluation will be conducted across the duration of the study, in parallel to the Phase 1 and Phase 2 activities. Data collection will involve a variety of ethnographic methods, including semi-structured interviews, observations and documentary analysis. Participants will include health professionals and patients as well as members of the trial team.

### Planned interventions

#### Phase 1: CBTI development methodology

The MRC revised their framework for the development of complex interventions [28], acknowledging that the process of development is circular and iterative, but that there are still distinct, if overlapping, stages that can be used to structure the intervention development process. The starting phase should be the establishment of the theoretical and empirical grounds of the intervention. This should involve a review of the extant evidence base and an identification or development of the theory on which the intervention will be based. They then recommend a modelling phase in which the nature of the intervention is specified. The key tasks of this stage are to identify the target problem, in our case fear of falling, the mechanisms whereby the proposed intervention will lead to change, and a specification of how this change will be measured. There is currently less consensus on how this part of the process should be enacted, but there is an agreement that the process of intervention development and implementation should be well documented enough to be independently replicable. The intervention should then be further refined and re-evaluated to ensure its effectiveness. The objectives for development of the CBTI were therefore:

1. To further review the evidence base for fear of falling.

2. To investigate patients’ experience of fear of falling using a cognitive behavioural model as an assessment framework in a series of patient interviews.

3. To use data gathered in these first two stages to model a theoretically structured and empirically grounded intervention for this condition, including explicit formulation of therapeutic targets, change processes and outcome evaluation.

4. To interview patients and clinic staff to explore the acceptability of the proposed intervention and to adapt the intervention accordingly.

5. To use the data from steps 3 and 4 to further develop and formalise the components of a short CBT training package for the intervention delivery staff.

6. To produce clinician and patient treatment manuals for the CBTI and establish the form of the treatment as usual arm, since this forms the control condition, with the intervention comprising the CBTI plus treatment as usual.

### Identification of existing theory and evidence base

From the existing literature (see ‘Background’ above) we already have some indications of falls and fear of falling epidemiology and patient characteristics, possible mechanisms involved in the predisposition to and maintenance of fear of falling, trials of psychosocial interventions in this condition and current service provision to this population. It is abundantly clear that fear of falling is a significant clinical problem, and those patients who experience it are also frequently anxious, depressed and activity avoidant.

### Patient interviews for CBTI development

The perspectives of patients are important for intervention development, in terms of understanding both (i) the appropriateness of the intervention for managing the clinical problem, and (ii) social and organisational factors that are likely to affect the patient’s uptake and involvement in the intervention. This part of the intervention development programme sought to further investigate patient characteristics, with a view to model development, by interviewing approximately 20 patients using a broad cognitive behavioural framework as an assessment tool. The cognitive behavioural model distinguishes between what made a person vulnerable to a problem (predisposing factors), what triggered the current problem (precipitating factors) and what is currently maintaining it (perpetuating factors). The model further distinguishes between physical, emotional, cognitive, behavioural and social factors in each domain. Finally, it looks for interactions between perpetuating factors (such as how anxious thoughts may increase physiological arousal, and vice versa). Such an approach resonates with Zijlstra and colleagues recent cognitive behavioural intervention aimed at improving the perception of falls and falls risks and increasing activity and safe behaviour [19]. Using this model as a template, we further investigated the relevant factors in the cause and maintenance of fear of falling.

Also in this CBTI developmental phase, and as part of the qualitative process evaluation, a set of 10 interviews were conducted with a different sample of patients, to focus explicitly on social and organisational factors likely to affect normalisation of the intervention from the perspective of patients.

### CBTI model development

Data from the literature and interviews described were used to further refine the extant models of fear of falling. On the basis of current knowledge [16-19], we would expect anxious cognitions to be maintaining activity avoidance; physical tension and anxious cognitions to be interfering with walking; underactivity to be maintaining physical weakness and loss of confidence and/or competence. Interventions were developed accordingly, targeting these maintaining factors or whatever else emerged from the preceding stages.

### Interviews with staff

As noted above, most of the literature cited notes the need for, and lack of, psychological service provision in this area. With a view to making our intervention as pragmatic and widely replicable as possible, we trained HCAs in the model and interventions derived from the outlined research. As previously noted [25,26,29] this is a complex process with many potential barriers and facilitators at the individual, interpersonal and institutional level. To maximise the possibility of the intervention’s uptake we utilised May’s Normalisation Process Theory [26,30]. This provides a framework for assessing the likelihood of a new intervention becoming normal practice, and for describing the process whereby it does so. During Phase 1, the qualitative process evaluation included a set of semi-structured interviews with key professionals involved in the development and delivery of the intervention (n = 5-7). This included clinical and other related professionals and provided us with a framework for predicting and increasing the likelihood of the intervention’s implementation.

### Putting the training and trial materials together

Our team has experience of training and supervising non-mental health professionals in psychosocial interventions [23] and has already evolved materials and methods, which were further refined for this staff and patient group. These materials are now being trialled in the randomised trial, as detailed below.

#### Phase 2: RCT of CBTI versus usual care

Patients are recruited in the falls clinics following appropriate identification of inclusion and exclusion criteria. They are then given study literature and an expression of interest form for return by post. A recruitment visit is then arranged, and informed written consent obtained. Patients are randomised in 1:1 ratio, with stratification by gender and referral to strength and balance training classes to CBTI plus treatment as usual, or control (treatment as usual) groups. CBTI sessions last approximately 45 minutes with 15 minutes of preparation time, and are based on an individualised formulation that identifies and targets the factors maintaining fear of falling for that individual. Treatment is weekly for 8 weeks, with a single reinforcement CBTI session 6 months after the last CBTI session. Follow-up will be for 12 months to assess the longevity of CBTI’s effects. Treatment as usual comprises the routine care that all patients at the falls clinic receive. All undergo detailed falls-oriented physiotherapy assessment, lying and standing blood pressure measurement, electrocardiography, bone health assessment using the FRAX™ [31] tool, Mini-Mental State Examination (MMSE) [32], 15-item Geriatric Depression Scale [33], FES-I [19] and visual acuity assessment [1]. A comprehensive overview with a falls expert, utilising the information gained from the other parts of the evaluation, rounds off the assessment.

### Inclusion/exclusion criteria

#### Inclusions

Consecutive community-dwelling patients aged 60 years and over, of both sexes, attending falls services in the North East of England with excessive or undue fear of falling as assessed by a FES-I score of >23. The original FES [12] and its internationally validated derivative scale the FES-I [20] were not designed as scales with rigidly defined numerical cut-off values, rather as tools to explore different aspects of falls and balance confidence in individual patients through the medium of self-efficacy evaluation. Use of the scales as tools for defining and quantifying fear of falling in the context of inclusion criteria for fear of falling RCTs has thus been controversial. Counterbalancing this is the FES-I’s undoubted clinical and research validity and utility, its validation in UK patient groups and its translation in multiple languages and cultures, allowing potential cross-cultural comparisons of data [7]. A number of studies have provided some useful data on cut-off scores to define those likely to have significant fear of falling. Ersoy *et al*. followed 125 women age 50 years and over for 6 months, comparing confidence and fear of falling measures in those who fell versus those who did not. An FES-I score of >26 was a potent predictor of future falls in those who fell (OR = 7.28, per additional point, 95% CI 2.25 to 23.61, *P* = 0.001) [34]. A much larger and more recent longitudinal study showed that an FES-I score of >23 on the 16-item version used routinely in our clinic (and in this study corresponded with high concern about falling) [35]. The authors, while sympathetic to the concerns expressed above, reasonably argued that intervention research in fear of falling was hampered by a dearth of responsive, valid and sensitive instruments and that their approach allows such use of the FES-I [35]. Accordingly we have chosen to use the cut-off of >23 for participant inclusion in our study.

#### Exclusions

Patients with cognitive impairment (MMSE <24) are excluded from the study given the paucity of data on the use of fear of falling measures in this patient group and the difficulties in conducting CBTIs in those with significant cognitive impairment. Patients with a life expectancy of <1 year and those requiring psychosocial interventions unrelated to fear of falling will also be excluded.

### Ethical and regulatory arrangements

Favourable ethical opinion from the Newcastle and North Tyneside 1 Research Ethics Committee (Phase 1 REC reference: 11/NE/0090 Phase 2 REC Reference: 12/NE/0006) has been obtained and subsequent Research and Development and Caldicott approvals have been granted to all parts of the study. The Newcastle upon Tyne Hospitals NHS Foundation Trust is the sponsor for the trial.

### Risks and anticipated benefits for trial participants and society

The potential risks associated with the STRIDE study are few. The main anticipated issue centres on the potential for patients to gain confidence and lose their fear of falling in a way that is inconsistent with their improvement (or lack thereof) in physical function. In other words, patients who have hitherto considerably limited activity through fear of falling have the potential to increase activity levels through amelioration of the condition with the CBTI before any physical interventions have been able to take effect and therefore to increase their risk of an actual fall. In practice, this is unlikely given the prolonged course of the CBTI. We believe the potential benefits to individuals and society in terms of increasing activity, avoidance of social isolation, enhancement of independence and avoidance of injuries and hospitalisations from examining the effectiveness of psychological interventions in people with fear of falling are, nevertheless, worth such risks.

### Sample size

#### Phase 1

Anxiety disorder tends to have a fairly narrow range of maintaining factors which, most usually, are catastrophic beliefs and activity avoidance. The psychological constructs of fear of falling suggest that a similar range of maintaining factors is likely to be in operation. We therefore expected the number of patients needed to consistently identify such factors to be relatively small; prior work from our group showed that saturation was reached with 12 [23], and will be no more than 30, interviews. Phase I was successfully completed with 15 such interviews.

#### Phase 2

Primary outcome is change in fear of falling (as measured by the FES-I) at 12 months. The estimated standard deviation is 12.5 [20]; the difference in group means that we wish to be able to detect is 4.0 (per clinical judgment in combination with observed effects in a range of studies) [18,35] corresponding to a standardized effect size of 0.32. Accordingly, the number required to provide full outcome data (80% power, 5% significance level) is 154 per group, or 308 in total. To allow for 25% drop-out (from previous experience in falls studies run locally with this patient group and from the CBT in fear of falling literature), we will recruit 412 subjects.

### Qualitative process evaluation (running across Phases 1 and 2)

Participants in the process evaluation will include members of the trial team (n = 10-15, each interviewed two to three times during the project), professionals involved in the delivery of the intervention (n = 5-7, interviewed at multiple points during the study) and patients who would be eligible for the CBTI (Phase 1 n = 10) and those receiving the intervention (Phase 2 n = 20).

### Statistical analysis

The primary analysis will be based on fear of falling (FES-I) assessed at baseline and 12 months. The change in fear of falling will be analysed using analysis of covariance. The dependent variable will be the FES-I score at 12 months; baseline FES-I will be included as a covariate. Stratification variables (gender, referral to strength and balance training classes) will be included as fixed effects. Potential differences between therapists will be investigated by fitting an additional random effect. Secondary outcomes will be assessed using similar methods.

### Effect of missing data

This is a comparatively frail population and we can expect some drop-out during the study. It is likely that those participants who are lost to follow-up during the trial may also be people with a tendency to experience poorer quality of life (in terms of fear of falling) than those who remain in the study. In this situation, the data cannot be considered to be missing at random. On the contrary, the event of a patient failing to complete the study may be informative. It is necessary to take into account any difference in drop-out rates and the non-randomness of the drop-out when comparing fear of falling per the FES-I between the two treatment groups. This will be done by jointly modelling ‘survival in the study’ and the repeated measures of fear of falling simultaneously [36] using software that has been developed as part of an MRC-funded programme of work (Grant G0400615; Statistical methodology for longitudinal studies in clinical research; Williamson PR, Diggle PJ and Henderson R). Time to drop-out will be analysed using a Cox proportion hazards model incorporating random effects. Fear of falling will be modelled using mixed models appropriate for repeated measures. A key feature of each of these models is that within each of them it is possible to fit a latent variable that can be conceptualised as the patient’s propensity to experience poor outcomes (both their likelihood to drop out of the study and their likelihood to have poorer FES-I scores). It is the inclusion of this latent variable that allows us to adjust our estimates of the treatment effect to allow for the different rates of drop-out in each group. Both models are estimated simultaneously; parameter estimates are based on maximising the joint likelihood over both the survival and repeated measures data.

### Outcome measures

#### Primary outcome measure

Change in fear of falling as measured by the FES-I [20].

#### Secondary outcome measures

##### Falls

a) Number of patients falling; b) number of falls; and c) fractures and significant soft tissue injuries: the close relationship between fear of falling and falls [5-20,37] means that measurements of falls and their adverse consequences are vital. Falls research is often hampered by poor definitions and hence difficult interpretation of falls outcome data. As per recent consensus guidelines [38], falls will be defined as ‘an unexpected event in which the participant comes to rest on the ground, floor, or lower level’. Participants and carers will be given verbal and written instructions to ‘. . . record each day in weekly-returned postage paid falls diaries any fall including a slip or trip in which you lost your balance and landed on the floor or ground or lower level’; weekly telephone prompts will be used to ensure contemporaneous reporting. We have previously used this technique successfully in similar populations, with published data showing in excess of 90% return rates [39,40].

### Domain-specific quality of life

Hospital Anxiety and Depression Scale (HADS): while fear of falling is the primary outcome measure, the inter-related nature of fear of falling and anxiety mean that change in anxiety is clinically highly relevant. In addition, it is vital to assess depression in this population [10]. The HADS, though widely used, was originally validated in a population with a maximum age of 65 years [41]. However, a recent large scale UK study in four centres across England, Wales and Scotland has shown that the scale’s psychometric constructs are intact and applicable to community-dwelling older people [42].

### Generic quality of life

World Health Organization Quality of Life questionnaire-older adults module (WHOQOL-OLD) [43], EuroQol 5-dimension scale (EQ-5D), the five-level version [44] and Short Form six-dimension health survey (SF-6D) [45]: the 24-item WHOQOL-OLD is a module of the World Health Organization’s broad measure of quality of life, the WHOQOL, which was designed for adults of all ages. The WHOQOL-OLD includes additional items specific to older people. The EQ-5D is a generic quality of life measure that has the added benefits of enabling cost utility analysis (below), as does the SF-6D.

### Social participation and social isolation

Social Disconnectedness and Perceived Isolation Scales [46,47]: avoidance of social contact and social isolation are crucial co-factors both caused by, and contributing to, fear of falling. Recent work from Cornwell *et al*. has shown that the Social Disconnectedness and Perceived Isolation scales [46,47], which measure both social isolation and participation, may be particularly useful in evaluating interventions in older people.

### Impact on function

Short Physical Performance Battery (SPPB) [48-50], functional reach [51], handgrip strength [52]: the SPPB is a well-validated set of lower limb performance tests (measures walking speed over the middle 8 feet of a 12-foot course at the participant’s own ‘usual speed’, a series of chair stands to assess muscle power, plus a test of balance), which has already been used in major surveys such as the Women’s Health and Aging [50]. Performed by one trained person, the SPPB takes 10 to 15 minutes to complete, with a composite score being derived by summing the category scores for each of the three tests. Functional reach is a good indicator of confidence in balance and increased risk of having a fall, while the measurement of maximum isometric handgrip strength (using a dynamometer) has functional relevance for supporting weight (for example holding on to a stair rail). It has been included in numerous surveys, and is predictive of both disability and mortality.

Primary and secondary outcome measures are performed by research assistants in the participants’ homes or at the falls clinic.

### Health economic evaluation

The economic evaluation will address the following critical resource allocation questions:

1. From the healthcare system perspective, what are the incremental costs and outcomes (FES-I) of treatment of usual falls service care that includes CBTI over usual falls service care usual among patients with a heightened fear of falling in the short term (12 months from the start of treatment, from the trial)?

2. From a more societal perspective, what are the incremental costs and quality-adjusted life years (QALYs) of the treatment options among patients with a heightened fear of falling in the short term (12 months from the start of treatment)?

The interpretation of the economic evaluation will crucially depend upon the clinical outcome of the trial [53]. For each of 1 and 2, should the therapy result in improved outcomes and cost savings relative to normal care, then the therapy would, according to the economic evaluation, be unambiguously recommended for adoption. Otherwise, should the treatment result in both increased costs and QALYs over normal care, the incremental cost per QALY gained will be calculated, thus showing the rate of return of QALYs gained for additional resources invested.

Stochastic sensitivity analysis will be undertaken to express the uncertainty around the incremental cost per life year gained/QALY ratio based on mean costs and outcomes [54-56]. An alternative way of looking at the above issues, however, is to say that the trial has been designed as a difference trial, and, therefore, we will attempt to estimate incremental cost per QALY, focusing on the joint density of costs and effects irrespective of their precision [57]. Results will be presented in the form of cost acceptability curves, thus using net benefit criteria [58].

To assess costs, data will be collected on:

1. Intervention and other outpatient expenditures: the costs for providing the therapy will be estimated on a per patient basis. This will be derived from the total costs of providing the therapy. This will include the training time for the falls service HCAs and any additional time and resources required over the eight weeks when the therapy is delivered, as well as the cost of initial evaluation for fear of falling. Data about levels of activity will also be used to inform sensitivity analyses.

2. Resource use outside of the falls service: trial participants will be asked, during the follow-up 12-month interviews of RCT participants, about their use of primary and secondary care and other community-based services. Again, use of such services will be costed using local rates of pay and prices. The interview schedule will also include questions on private costs incurred by patients, including over-the-counter expenses and the cost of accessing medical care, such as travel expense and lost time from leisure and work activities in those who are still economically active. We also include questions about the amount of informal care provided by friends and family of people with a heightened fear of falling. The cost of this care will be estimated from equivalent professional care providers pay when applicable and by the human capital approach [59].

To generate a cost per QALY*,* data on ‘utility’ or quality of life are required in combination with data on survival. Quality-of-life data will be gathered primarily using the EQ-5D [60,61]. The EQ-5D is a generic, preference-based measure of quality of life in which ‘health’ is defined in terms of five broad dimensions. Patients will be asked to rate their health status in terms of each of these dimensions every six months (when visiting clinics for follow-up). In the UK, health states in the EQ-5D classification have been allocated scores derived from a survey of values by members of the general public (where 0 = death and 1 = full health) [62]. The SF-6D will also be used to translate outcomes measured by the SF-36 into health state utilities using of a recently produced ‘tariff’ [45]. The requisite self-response items for these quality-of-life measures will be gained through self-completion questionnaires unless the patient requires support in which case they are interviewer administered during routine visits. In the case of failure to return, these data are collected by telephone interview. As quality-of-life data are not collected on a continuous basis, power curves (simply, a form of extrapolation) can be fitted to model quality-of-life profiles for the interventions groups from baseline to end of follow-up [45]. These power curves will take account of differences in survival between the groups, so permitting generation of a QALY for each mode of care and, more importantly, a QALY difference between them.

In addition to the above, we propose to better estimate the effect on quality of life of a fear of falling. Following validation of the scale by factor analysis [63] to ensure that it adequately represents concerns about falling during social and physical activities, we will estimate the effect on quality of life of a fear of falling. We will do this by exploring the degree to which any discrepancy between EQ-5D tariff values and self-reported quality of life are explained by a fear of falling. Should the FES-I be unsuitable, we will attempt to estimate the effect of a fear of falling directly from patients by means of a standard gamble or similar preference elicitation method.

### Qualitative process evaluation of the trial

The purpose of the process evaluation is to understand the organizing processes that underpin delivery of the trial and its reception by professionals and patients, we will undertake an ethnographic sub-study within the trial. May and colleagues have employed these methods previously [64-66]. The aim of the study is therefore to identify, describe, and explain the professional and organizational factors that promote or inhibit the implementation and integration of the intervention.

Throughout the study we will track the activities of key personnel and their interactions with each other as the trial develops. We will examine the implementation of the intervention by a rolling programme of interviews with members of the trial team (maximum n = 15), along with observations of team and other relevant meetings (n = 9 approximately), that focus on the delivery and take up of the intervention by participating health professionals and service users. We will interview all professionals involved in intervention development and service delivery (n = 7 approximately) including clinical and related professionals who will be able to contribute different perspectives. Professionals will be interviewed at multiple time points across the duration of the study, but we will utilise brief follow-up telephone interviews of these individuals to explore how the evaluation and interpretations of the intervention changes over time without overburdening individuals. The range, flexibility and depth of ethnographic research is a significant advantage in such work, and represents the only way such a project could be undertaken. We will interview a purposive sample of patients (n = 10 during Phase 1; n = 20 during Phase 2) focusing on their views/experiences of the intervention and factors affecting normalisation from their perspective. In all cases, interviews and observations will be undertaken only when informed consent has been obtained and recorded from participating researchers, patients, and health professionals. To reduce the burden of research, interviews with participating patients will not exceed 45 minutes duration without their express permission. All interviews will take place at a time and location of the interviewee’s choosing. Meetings and interviews will be audio recorded using digital voice recorders. Audio recordings will be transcribed, checked, and edited to ensure participants’ anonymity (with particular attention to removing identifying data from professionals’ transcripts). Transcripts will be stored in password-protected computer systems, and non-anonymised voice recordings or transcripts will be handled only by members of the research team and transcribers who have signed appropriate confidentiality agreements. We will use Normalization Process Theory as a conceptual framework from which to develop a structured qualitative analysis of the transcript data set using the Framework method pioneered by Ritchie and Spencer [67]. This analysis will lead to a robust conceptual model of the factors that have affected the course of the intervention. This model will be of value to other clinicians and researchers wishing to deploy the intervention since it will provide a guide to implementation, embedding, and integration in everyday clinical practice.

### Outcome data collection

Primary and secondary outcomes will be measured at baseline (pre-randomisation), at 8 weeks (that is at the end of CBTI) and 6 and 12 months post-randomisation. Assessment will also be made of harms, level of social care and dependency. Weekly symptom diaries (validated in similar populations [39,68]), completed and submitted weekly for 12 months, will record falls and injuries as described previously. Data will be collected for the purpose of the process evaluation on an ongoing basis over the course of the project.

### Research governance

#### Sponsor

Newcastle Hospitals NHS Foundation Trust is the trial sponsor and the study was adopted by the Northumberland, Tyne and Wear Comprehensive Local Research Network.

### Trial steering committee (TSC)

A trial steering committee comprising an independent topic area expert (Professor J Mason, Durham University) to act as chair of this important committee. Along with SWP (chief investigator (CI)), the study statistician (NS) and trial manager (CM), we also invited an independent falls expert (Dr C Bailey, Northumbria University), an independent medical statistician (Dr C Ramsay, Aberdeen University) and lay representative (Mrs A Watt, Age UK North Tyneside). Observers from the National Institute of Health Research Health Technology Assessment (NIHR HTA) programme (the funding body) are invited to all TSC meetings.

### Data monitoring and ethics committee (DMEC)

An independent chair (Professor RA Kenny, Dublin) leads the DMEC, with other members including an experienced trial statistician (Dr S Lewis), and a clinician with an interest in falling (Professor D Skelton, Glasgow Caledonian University) who are not part of the trial. The DMEC reports to the TSC and (via the TSC) to the HTA programme. Adverse events amongst study participants are collected and classified with respect to seriousness, causality and expectedness; the assessment of adverse events is made in the by the CI, and ultimately to the clinical members of the DMEC.

### Project timetable

Study start date: 1 June 2011

Phase 1: 10 months, to 31 March.2012

Phase 2: Participant recruitment: to 28 February 2014, final follow-up 31 January 2015.

Data analysis and write up: 3 months.

End of study: 30 April 2015*.*

Process evaluation data collection and analysis are conducted as appropriate at key stages throughout Phases 1 and 2 of the project.

## Discussion and trial status

Phase I of the study was completed successfully, with the development of training materials and successful recruitment and training of the HCAs in delivering the CBTI to time and target. Despite pre-study qualitative work suggesting an appropriate timetable for recruitment, Phase II began to run into recruitment problems from an early stage. A recovery plan was put in place following qualitative interviews with potential study participants, actual participants, carers and staff members to identify barriers and promoters of recruitment. With the assistance of the TSC and agreement with the DMEC and the funder, NIHR HTA, alongside appropriate permissions and approvals, two further sites to the original single study site were brought on board. A 7-month extension to the study was granted, with the desired outcome of the appropriate recruitment target within reach at time of writing.

The CBTI has uncovered interesting insights into fear of falling that will be of use to researchers trying to improve this nebulous and limiting condition regardless of the outcome of the RCT. The use of non-mental health practitioners in this role has been studied previously with our experience in this study reinforcing the potential for suitably trained and supervised HCA assistant grade staff to perform such therapies. This has clear implications for cash-strapped health providers with a relatively small base of clinical psychologists and therapists available to manage such ubiquitous clinical problems. Similarly, the process evaluation has already yielded important observations that will be of use to researchers and those implementing healthcare innovations.

## Abbreviations

CBT: cognitive behavioural therapy; CBTI: cognitive behavioural therapy-based intervention; DMEC: data monitoring and ethics committee; EQ-5D: Euroquol 5-dimension health questionnaire; FES: Falls Efficacy Scale; FES-I: Falls Efficacy Scale - International version; HADS: Hospital Anxiety and Depression Scale; HCA: healthcare assistant; MMSE: Mini-Mental State Examination; NIHR HTA: National Institute of Health Research Health Technology Assessment; QALY: quality-adjusted life year; RCT: randomised controlled trial; SPPB: Short Physical Performance Battery; SF-6D: Short Form 6-dimension health survey; TSC: trial steering committee; WHOQOL-OLD: WHO Quality of Life questionnaire-older adults module.

## Competing interests

The authors declare that they have no competing interests.

## Authors’ contributions

SWP, VD, EM, TF, PM, JO’B, NSt, NSa, AC, CM, SW and CB contributed to the conception and design, manuscript writing and final approval of the manuscript. All authors read and approved the final manuscript.

## Authors’ information

SWP is a senior lecturer and consultant geriatrician with extensive experience in falls from both clinical and research perspectives. VD is a clinical psychologist and senior lecturer in psychology with experience of both cognitive behavioural therapy, and its implementation by non-mental health professionals. NSa is a clinical psychologist with particular expertise in the older patient. EM is director of the Newcastle Clinical Trials Unit while NSt is senior research associate in statistics with extensive experience of falls-related randomised controlled trials. TF and CB are highly experienced qualitative researchers with particular expertise in process normalization theory, while PM is a senior research associate in health economics with extensive experience of falls health economic evaluation. AC is chief executive of North Tyneside Age UK, while JO’B is professor of old age psychiatry with extensive experience of clinical research in the older patient with mental health problems. SW is an expert in balance confidence issues affecting the older person, with particular clinical and research expertise in function measures of physical performance in older age, while CM is the trial manager who contributed to protocol writing and design.

## References

[B1] American Geriatrics Society, British Geriatrics Society, and American Academy of Orthopedic Surgeons Panel on Falls Prevention Guideline for the prevention of falls in older persons J Am Geriatr Soc 2001 49 664 672 10.1046/j.1532-5415.2001.49115.x 11380764

[B2] Painter JA Elliott SJ Hudson S Falls in community-dwelling adults aged 50 years and older: prevalence and contributing factors J Allied Health 2009 38 201 207 20011818

[B3] Scuffham P Chaplin S Legood R Incidence of costs of unintentional falls in older people in the United Kingdom J Epidemiol Community Health 2003 57 740 744 10.1136/jech.57.9.740 12933783 PMC1732578

[B4] Heinrich S Rapp K Rissmann U Becker C König HH Cost of falls in old age: a systematic review Osteoporos Int 2010 21 891 902 10.1007/s00198-009-1100-1 19924496

[B5] Vellas BJ Wayne SJ Romero LJ Baumgartner RN Garry PJ Fear of falling and restriction of mobility in elderly fallers Age Ageing 1997 26 189 193 10.1093/ageing/26.3.189 9223714

[B6] Murphy SL Williams CS Gill TM Characteristics associated with fear of falling and activity restriction in community-living older persons J Am Geriatr Soc 2002 50 516 520 10.1046/j.1532-5415.2002.50119.x 11943049 PMC3046411

[B7] Zijlstra GA Van Haastregt JCM Van Rossum E Van Eijk JTM Yardley L Kempen GIJM Interventions to reduce fear of falling in community-living older people: a systematic review J Am Geriatr Soc 2007 55 603 615 10.1111/j.1532-5415.2007.01148.x 17397441

[B8] Zijlstra GA Van Haastregt JC Van Eijk JT Van Rossum E Stalenhoef PA Kempen GI Prevalence and correlates of fear of falling, and associated avoidance of activity in the general population of community-living older people Age Ageing 2007 36 304 309 10.1093/ageing/afm021 17379605

[B9] Scheffer AC Schuurmans MJ Van Dijk N van der Hooft T De Rooij SE Fear of falling: measurement strategy, prevalence, risk factors and consequences among older persons Age Ageing 2008 37 19 24 18194967 10.1093/ageing/afm169

[B10] Van Haastregt JC Zijlstra GA Van Rossum E Van Eijk JT Kempen GI Feelings of anxiety and symptoms of depression in community-living older persons who avoid activity for fear of falling Am J Geriatr Psychiatry 2008 16 186 193 10.1097/JGP.0b013e3181591c1e 18310549

[B11] Jorstad EC Hauer K Becker C Lamb SE on behalf of the ProFaNE Group Measuring the outcomes of fear of falling: a systematic review J Am Geriatr Soc 2005 53 501 510 10.1111/j.1532-5415.2005.53172.x 15743297

[B12] Tinetti ME Richman D Powell L Falls efficacy as a measure of fear of falling J Gerontol 1990 45 P239 P243 10.1093/geronj/45.6.P239 2229948

[B13] Reelick MF Van Iersel MB Kessels RP Rikkers MG The influence of fear of falling on gait and balance in older people Age Ageing 2009 38 435 440 10.1093/ageing/afp066 19451658

[B14] Deshpande N Metter EJ Lauretani F Bandinelli S Guralnik HJ Ferrucci L Activity restriction induced by fear of falling and objective and subjective measures of physical function: a prospective cohort study J Am Geriatr Soc 2008 56 615 620 10.1111/j.1532-5415.2007.01639.x 18312314 PMC2645621

[B15] Austin N Devine A Dick I Prince R Bruce D Fear of falling in older women: a longitudinal study of incidence, persistence and predictors J Am Geriatr Soc 2007 55 1598 1603 10.1111/j.1532-5415.2007.01317.x 17908062

[B16] BonnerupVind A Elkjaer Andersen H Damgaard Pedersen K Joergensen T Schwarz P The effect of a program of multifactorial fall prevention on health-related quality of life, functional ability, fear of falling and psychological well-being. A randomized controlled trial Aging Clin Exp Res 2010 22 249 254 10.1007/BF03324804 19934621

[B17] Tennstedt S Howland J Lachman M Peterson E Kasten L Jette A A randomized, controlled trial of a group intervention to reduce fear of falling and associated activity restriction in older adults J Gerontol: Psychological Sciences 1998 53B P384 P392 10.1093/geronb/53B.6.P384 9826971

[B18] Clemson L Cumming RG Kendig H Swann M Heard R Taylor K The effectiveness of a community-based program for reducing the incidence of falls in the elderly: A randomized trial J Am Geriatr Soc 2004 52 1487 1494 10.1111/j.1532-5415.2004.52411.x 15341550

[B19] Zijlstra GA Van Haastregt JCM Ambergen T Van Rossum E Van Eijk JTM Tennstedt SL Kempen GIJM Effects of a multicomponent cognitive behavioural group intervention on fear of falling and activity avoidance in community-dwelling older adults: Results of a randomized controlled trial J Am Geriatr Soc 2009 57 2020 2028 10.1111/j.1532-5415.2009.02489.x 19793161

[B20] Yardley L Beyer N Hauer K Kempen G Piot-Ziegler C Todd C Development and initial validation of the Falls Efficacy Scale-International (FES-I) Age Ageing 2005 34 614 619 10.1093/ageing/afi196 16267188

[B21] Williams C Garland A A cognitive-behavioural therapy assessment model for use in everyday clinical practice Adv Psychiatr Treat 2002 8 172 179 10.1192/apt.8.3.172

[B22] Kennedy T Jones R Darnley S Seed P Wessely S Chalder T Cognitive behaviour therapy in addition to antispasmodic treatment for irritable bowel syndrome in primary care: randomised controlled trial BMJ 2005 331 435 437 10.1136/bmj.38545.505764.06 16093252 PMC1188111

[B23] Daniilidou P Carding P Wilson J Drinnan M Deary V Cognitive behavioural therapy for functional dysphonia: a pilot study Ann Otol Rhinol Laryngol 2007 116 723 730 17987776 10.1177/000348940711601002

[B24] Campbell NC Murray E Darbyshire J Emery J Farmer A Griffiths F Guthrie B Lester H Wilson P Kinmonth AL Designing and evaluating complex interventions to improve health care BMJ 2007 334 455 459 10.1136/bmj.39108.379965.BE 17332585 PMC1808182

[B25] Oakley A Strange V Bonell C Allen E Stephenson J RIPPLE Study Team Health services research: process evaluation in randomised controlled trials of complex interventions BMJ 2006 332 413 416 10.1136/bmj.332.7538.413 16484270 PMC1370978

[B26] May C Finch T Implementation, embedding, and integration: an outline of Normalization Process Theory Sociology 2009 43 535 554

[B27] Lunde LH Nordhus IH Pallesen S The effectiveness of cognitive and behavioural treatment of chronic pain in the elderly: a quantitative review J Clin Psychol Med Settings 2009 16 254 262 10.1007/s10880-009-9162-y 19424781

[B28] Craig P Dieppe P Macintyre S Michie S Nazareth I Petticrew M Medical Research Council Guidance Developing and evaluating complex interventions: the new Medical Research Council guidance BMJ 2008 337 a1655 10.1136/bmj.a1655 18824488 PMC2769032

[B29] Greenhalgh T Robert G Macfarlane F Bate P Kyriakidou O Diffusion of innovations in service organizations: systematic review and recommendations Milbank Q 2004 82 581 629 10.1111/j.0887-378X.2004.00325.x 15595944 PMC2690184

[B30] May C A rational model for assessing and evaluating complex interventions in health care BMC Health Serv Res 2006 6 86 10.1186/1472-6963-6-86 16827928 PMC1534030

[B31] Kanis JA Johnell O Oden A Johansson H McCloskey E FRAX and the assessment of fracture probability in men and women from the UK Osteoporosis Int 2008 19 385 397 10.1007/s00198-007-0543-5 PMC2267485 18292978

[B32] Folstein MF Folstein SE McHugh PR “Mini-mental state”. A practical method for grading the cognitive state of patients for the clinician J Psychiatr Res 1975 12 189 198 10.1016/0022-3956(75)90026-6 1202204

[B33] Sheikh JI Yesavage JA Geriatric Depression Scale (GDS) Brink TL Recent evidence and development of a shorter version Clinical gerontology: a guide to assessment and intervention 1986 New York: The Haworth Press 165 173

[B34] Ersoy Y Macwalter RS Durmus B Altay ZE Baysal O Predictive effects of different clinical balance measures and the fear of falling on falls in postmenopausal women aged 50 years and over Gerontology 2009 55 660 665 10.1159/000235652 19690394

[B35] Delbaere K Close JCT Mikolaizak AS Sachdev PS Brodaty H Lord SR The Falls Efficacy Scale International (FES-I). A comprehensive longitudinal validation study Age Ageing 2010 39 210 216 10.1093/ageing/afp225 20061508

[B36] Henderson R Diggle PJ Dobson A Joint modelling of longitudinal measurements and event time data Biostatistics 2000 1 465 480 10.1093/biostatistics/1.4.465 12933568

[B37] Cameron ID Staford B Cumming RG Birks C Kurrle SE Lockwood K Quine S Finnegan T Salkeld G Hip protectors improve falls self-efficacy Age Ageing 2000 29 57 62 10.1093/ageing/29.1.57 10690697

[B38] Lamb SE Jorstad-Stein EC Hauer K Becker C Prevention of Falls Network Europe and Outcomes Consensus Group Development of a common outcome data set for fall injury prevention trials: the Prevention of Falls Network Europe consensus J Am Geriatr Soc 2005 53 1618 1622 10.1111/j.1532-5415.2005.53455.x 16137297

[B39] Parry SW Bexton RS Steen N Kenny RA Pacing in elderly recurrent fallers with carotid sinus hypersensitivity (PERF- CSH): a randomised, double-blind, placebo controlled cross-over trial Heart 2009 95 405 409 19124530 10.1136/hrt.2008.153189

[B40] Kenny RA Richardson DA Bexton RS Steen N Bond J Carotid sinus syndrome: a modifiable risk factor for nonaccidental falls in older adults (SAFE PACE) J Am Coll Cardiol 2001 38 1491 1496 10.1016/S0735-1097(01)01537-6 11691528

[B41] Zigmond AS Snaith RP The hospital anxiety and depression scale Acta Psychiatr Scand 1983 67 370 10.1111/j.1600-0447.1983.tb09716.x 6880820

[B42] Gale R Allerhand M AihieSayer A Cooper C Dennison EM Starr JM Ben-Shlomo Y Gallacher JE Kuh D Deary IJ the HALCyon Study Team The structure of the hospital anxiety and depression scale in four cohorts of community-based, healthy older people: the HALCyon Programme Int J Geriatric Psych 2010 22 559 571 10.1017/S1041610210000256 20214846

[B43] Power M Quinn K Schmidt S WHOQOL-OLD Group Development of the WHOQOL-old module Qual Life Res 2005 14 2197 2214 10.1007/s11136-005-7380-9 16328900

[B44] The EuroQol Group EuroQol: a new facility for the measurement of health-related quality of life Health Policy 1990 16 199 208 10109801 10.1016/0168-8510(90)90421-9

[B45] Brazier JE Roberts JR Deverill M The estimation of a preference-based measure of health from the SF-36 J Health Econ 2002 21 271 292 10.1016/S0167-6296(01)00130-8 11939242

[B46] Cornwell EY Waite LJ Social disconnectedness, perceived isolation, and health among older adults J Health Soc Behav 2009 50 31 48 10.1177/002214650905000103 19413133 PMC2756979

[B47] Cornwell EY Waite LJJ Measuring social isolation among older adults using multiple indicators from the NSHAP study J Gerontol B Psychol Sci Soc Sci 2009 64 Suppl 1 i38 i46 19508982 10.1093/geronb/gbp037 PMC2800811

[B48] Guralnik JM Simonsick EM Ferrucci L Glynn RJ Berkman LF Blazer DG Scherr PA Wallace RB A short physical performance battery assessing lower extremity function: association with self-reported disability and prediction of mortality and nursing home admission J Gerontol 1994 49 M85 M94 10.1093/geronj/49.2.M85 8126356

[B49] Bandinelli S Lauretani F Boscherini V Gandi F Pozzi M Corsi AM Bartali B Lova RM Guralnik JM Ferrucci L A randomized, controlled trial of disability prevention in frail older patients screened in primary care: the FRASI study. Design and baseline evaluation Aging Clin Exp Res 2006 18 359 366 10.1007/BF03324831 17167299 PMC2659809

[B50] Simonsick EM Guralnik JM Volpato S Balfour J Fried LP Just get out the door! Importance of walking outside the home for maintaining mobility: findings from the Women’s Health and Aging Study J Am Geriatr Soc 2005 53 198 203 10.1111/j.1532-5415.2005.53103.x 15673341

[B51] Duncan PW Studenski S Chandler J Prescott B Functional reach: predictive value in a sample of elderly male veterans J Gerontol 1992 47 M93 M97 10.1093/geronj/47.3.M93 1573190

[B52] Rantanen T Era P Heikkinen E Maximal isometric strength and mobility among 75-year-old men and women Age Ageing 1994 23 132 137 10.1093/ageing/23.2.132 8023721

[B53] Donaldson C Hundley V McIntosh E Using economics alongside clinical trials: why we cannot choose the evaluation technique in advance Health Econ Lett 1996 5 267 269 10.1002/(SICI)1099-1050(199605)5:3<267::AID-HEC209>3.0.CO;2-X 8817300

[B54] Stinnett AA Mullahy J Net health benefits: a new framework for the analysis of uncertainty in cost effectiveness analysis Med Decis Mak 1998 18 S68 S80 10.1177/0272989X9801800209 9566468

[B55] Briggs AH A Bayesian approach to stochastic cost-effectiveness analysis Health Econ 1999 8 257 261 10.1002/(SICI)1099-1050(199905)8:3<257::AID-HEC427>3.0.CO;2-E 10348420

[B56] Van Hout BA Al MJ Gordon GS Rutten FF Costs, effects and C/E ratios alongside a clinical trial Health Econ 1994 3 309 319 10.1002/hec.4730030505 7827647

[B57] Briggs AH O’Brien BJ The death of cost-minimisation analysis? Health Econ 2001 10 179 184 10.1002/hec.584 11252048

[B58] Briggs AH O’Brien BJ Blackhouse G Thinking outside the box: recent advances in the analysis and presentation of uncertainty in cost-effectiveness studies Annu Rev Public Health 2002 23 377 401 10.1146/annurev.publhealth.23.100901.140534 11910068

[B59] Drummond M O’Brien B Stoddart G Torrance G Methods for the economic evaluations of health care programmes 1997 2 Oxford: Oxford University Press

[B60] Kind P Spiker B The EuroQol instrument: an index of health-related quality of life Quality of life and pharmacoeconomics in clinical trials 1996 2 Philadelphia: Lippincott-Raven Publishers

[B61] Brooks R EuroQol: the current state of play Health Policy 1996 37 53 72 10.1016/0168-8510(96)00822-6 10158943

[B62] Dolan P Gudex C Kind P Williams A A social tariff for the EuroQol: results from a UK general population survey. Centre for Health Economics, Discussion Paper 138 1995 York: Centre for Health Economics, University of York

[B63] Hurley AE Scandura TA Schriesheim CA Brannick MT Seers A Vandenberg RJ Williams LJ Exploratory and confirmatory factor analysis: guidelines, issues, and alternatives J Organ Behav 1997 18 667 683 10.1002/(SICI)1099-1379(199711)18:6<667::AID-JOB874>3.0.CO;2-T

[B64] Robson SC Kelly T Howel D Deverill M Hewison J Lie ML Stamp E Armstrong N May CR Randomised preference trial of medical versus surgical termination of pregnancy less than 14 weeks’ gestation (TOPS) Health Technol Assess 2009 13 1 124 iii-iv 19906334 10.3310/hta13530

[B65] Mort M Williams T Mair F Gask L Health technology assessment in its local contexts: studies of telehealthcare Soc Sci Med 2003 57 697 710 10.1016/S0277-9536(02)00419-7 12821017

[B66] May CR Mair FS Dowrick CF Finch TL Process evaluation for complex interventions in primary care: understanding trials using the normalization process model BMC Fam Pract 2007 8 42 10.1186/1471-2296-8-42 17650326 PMC1950872

[B67] Ritchie J Spencer L Bryman A, Burgess R Qualitative data analysis for applied policy research Analysing Qualitative Data 1994 London: Routledge 173 194

[B68] Parry SW Steen N Galloway SR Kenny RA Bond J Falls and confidence related quality of life outcome measures in an older British cohort Postgrad Med J 2001 77 103 108 10.1136/pmj.77.904.103 11161077 PMC1741890

